# Effectiveness of physiotherapy techniques on depressive symptoms in older adults: a systematic review

**DOI:** 10.3389/fpubh.2025.1671788

**Published:** 2025-09-17

**Authors:** José Lesmes Poveda-López, Carolina Jiménez-Sánchez, Juan Francisco Roy, Raquel Lafuente-Ureta, Ana Aguilera-Gonzalo

**Affiliations:** ^1^Department of Physiotherapy, Faculty of Health Sciences, Universidad San Jorge, Zaragoza, Spain; ^2^Department of Psychology, Faculty of Health Sciences, Universidad San Jorge, Zaragoza, Spain; ^3^Department of Psychology, Faculty of Health Sciences, Universidad Internacional de La Rioja, Logroño, Spain

**Keywords:** physiotherapy, depression, older adult, effectiveness, systematic review

## Abstract

**Introduction:**

The older adult population suffers from a high prevalence of depression, representing an increasing burden on healthcare systems. In this context, this systematic review evaluated the effectiveness of physiotherapy for the management of depressive symptoms in this population. The population aged 65 and over is increasing in developed countries such as Spain, currently accounting for 19.09% and projected to reach 25.2% within the next decade. Depression is one of the most prevalent mental health conditions in this group, affecting 5% of community-dwelling individuals and 25% in institutional settings, significantly reducing quality of life and increasing the risk of dementia. While the pharmacological and psychotherapeutic treatments are standard, they present adverse effects and limitations in this population, prompting the exploration of physiotherapy as a non-pharmacological alternative.

**Objective:**

To analyze the scientific evidence regarding the effectiveness of combining conventional treatments with physiotherapy techniques for symptomatic improvement in older patients with depression, and to identify the most effective physiotherapy technique, when combined with conventional treatment, for depressive symptoms in older adults.

**Methods:**

We conducted a systematic review following PRISMA guidelines, and we performed searches in PubMed, Web of Science (WOS), Scopus and Cochrane Library databases. Study quality and risk of bias were assessed using the Cochrane Collaboration’s tool.

**Results:**

Eight randomized controlled trials, encompassing a total of 1,368 older participants diagnosed with depression or depressive symptoms who received physiotherapy, were included. The findings indicate that physiotherapy techniques, particularly therapeutic exercise, in combination with conventional treatments, may improve depressive symptoms in this population.

**Conclusion:**

Although we observed positive trends in the effectiveness of physiotherapy for depression in older adults, further research is required to validate its clinical efficacy and inform evidence-based treatment guidelines.

**Systematic review registration:**

https://www.crd.york.ac.uk/PROSPERO/view/CRD420251079161.

## 1 Introduction

Global demographic shifts are reshaping contemporary societies, with a notable rise in the population aged 65 and over, particularly in countries like Spain, where life expectancy is among the highest in the world, exceeding 83 years according to recent epidemiological studies (1). Currently, older adults represent approximately 20.3% of the Spanish population, and projections estimate that this figure will increase to around 25% by 2035 (2). This demographic trend has been accompanied by a growing prevalence of chronic conditions among older adults, including hypertension (30–45%), type 2 diabetes (10–20%), osteoarthritis (20–30%), and depression (5–20%) (3).

Depression is one of the most prevalent mental health disorders affecting approximately 5% of older adults living in the community and up to 25% of those in institutional settings (4). In this population, depression is frequently associated with chronic illnesses, functional impairment, cognitive decline, and a reduced health-related quality of life. The consequences are profound; depression in older adults significantly increases the risk of mortality and is a major contributor to the elevated suicide rates observed in this age group (5). Furthermore, depression is closely linked to cognitive deterioration and is considered a risk factor for the development of dementias, including Alzheimer’s disease (6).

In older adults, the standard treatment for depression usually involves a combination of psychotherapy (7) and antidepressant medications, with medication being the more commonly used approach. However, these approaches present notable limitations and risks within this population (8). Psychotherapy can be expensive and require sustained engagement from patients, which may be challenging for some older individuals. Meanwhile, antidepressant medications are associated with side effects such as drowsiness, nausea, and dizziness, and they may interact adversely with other medications commonly used to manage age-related comorbidities (9, 10). These factors can significantly hinder treatment adherence and compliance. Given these challenges and the potential adverse effects of pharmacological interventions, the exploration and implementation of non-pharmacological alternatives for treating depression in older adults is of critical importance (11–13).

Although the link between mental and physical health is well recognized, the impact of physical health on mental well-being remains underexplored (14). In this context, physiotherapy emerges as a promising non-pharmacological intervention (15), having been shown to improve mental health outcomes in the general population, —enhancing mood, reducing stress and anxiety, and promoting overall quality of life (16, 17). Furthermore, within the field of physiotherapy for mental health (16), several interventions—such as specific physical exercise programs, body-based programs, manual therapy, and stretching techniques—have shown promising results in improving psychological well-being (18–21). However, while these benefits are well-documented in broader populations, the specific evidence regarding the effectiveness of physiotherapy techniques in alleviating depressive symptoms among older adults remains limited and fragmented (22).

Therefore, this systematic review aims to evaluate the existing scientific evidence on the effectiveness of physiotherapy interventions as an adjunctive treatment for depression in older adults, present current clinical practice findings, and contribute the developing evidence-based guidelines for this population.

## 2 Methods

This systematic review was conducted by following the Preferred Reporting Items for Systematic Reviews and Meta-Analyses (PRISMA) statement (23) (see Supplementary File S1 for PRISMA checklists). We registered the review protocol in PROSPERO (International Prospective Register of Systematic Reviews) (number CRD420251079161).

### 2.1 Eligibility criteria and structured question formulation

This systematic review’s inclusion and exclusion criteria were established based on the PICOS (Participants, Interventions, Comparison, Outcomes, and Study Design) framework and are detailed in Supplementary File S2.

The research question was formulated before the literature review: Are physiotherapy techniques combined with conventional treatment effective in improving depressive symptoms in older adult patients?

### 2.2 Search strategy for study identification

We conducted an exhaustive search of scientific literature across the major biomedical databases: PubMed, Web of Science (WOS), Scopus, and Cochrane Library. The search was performed up to June 30, 2025. Two independent researchers conducted the search, reaching the same results, and filtered for articles published in Spanish, English, and French. No other filters or limits based on date or study type were applied. The same two researchers searched manually using appropriate terms and following the specific search procedure for each database (see Supplementary File S3). Search terms were developed by combining MeSH (Medical Subject Headings) terms and free-text keywords (see Supplementary File S4).

### 2.3 Data selection and collection process

We exported identified studies from the databases to the ZOTERO reference management tool to remove duplicates. Subsequently, article titles and abstracts were independently screened manually into an Excel spreadsheet by two reviewers (J.L.P.L. and A.A.G.) to identify potentially eligible studies. In disagreement, consensus was reached through discussion or involving a third reviewer (R.L.U.). Articles that met the initial criteria were retrieved in full text for more detailed evaluation. The final eligibility assessment of the full text was also performed independently by the two reviewers, applying the PICOS criteria.

### 2.4 Variables and characteristics of included studies

For each included study, two reviewers extracted the following data:

- Lead author and year of publication.
- Study design (confirm RCT).
- Participants’ demographic characteristics (mean age, sex, depression diagnosis).
- Physiotherapy intervention characteristics (type of technique, duration, frequency, intensity, supervision).
- Control group characteristics (type of intervention, duration).
- Measurement scales used to assess depressive symptoms (with cutoff points if relevant).
- The main outcomes related to the improvement of depressive symptoms.

In addition to the aforementioned variables, we considered the intervention setting differently between community/home-based and geriatric care environments. Furthermore, specific intervention characteristics were recorded, including the type of physiotherapy, intervention duration, number of sessions, periodicity, and participant adherence.

We paid particular attention to measures used to assess the outcome variable, namely, the symptoms or severity of depression. From these measures, the baseline score (pre-intervention) and the difference between pre- and post-intervention scores were documented, facilitating a direct comparison between the control and intervention groups.

We extracted systematically detailed key information from individual studies such as the author/year, intervention type, duration, sample size, main outcomes, and risk of bias.

Given the high clinical and methodological heterogeneity observed across the included studies (manifested in differences in interventions, disease severity, and outcome measures), a structured narrative synthesis was performed, furthermore interventions were not grouped by type. Instead, this strategy allowed for identifying relevant patterns and consistencies within the literature without presuming non-existent statistical homogeneity.

We justified the adoption of this methodological approach by the diversity in study designs and the absence of comparable statistical data in numerous instances.

Although a formal heterogeneity analysis (meta-regression or statistical subgroup analysis) wasn’t conducted, potential sources of variation were explored through narrative comparisons among study subgroups. In this sense, we examined actors such as risk of bias, intervention type, severity of the condition, and setting (institutionalized vs. non-institutionalized). These comparisons are discussed in the discussion and limitations section, allowing for the identification of potential moderators of intervention effectiveness.

### 2.5 Assessment of certainty in evidence

We applied the Grading of Recommendations Assessment, Development and Evaluation (GRADE) (24) methodology to assess the certainty of the evidence for the outcome of depression. This framework allowed for a systematic evaluation of the confidence in the body of evidence for this specific outcome across all included studies. All relevant information about this certainty assessment can be found in the Results section.

### 2.6 Assessment of study quality and risk of bias

The methodological quality and risk of bias of the included studies were independently assessed by the two reviewers, who conducted a meticulous risk of bias assessment for each study included in our analysis using the Cochrane Rob-2 (25). The reviewers independently undertook each study’s evaluation, with discrepancies resolved through discussion or consulting a third reviewer when necessary. This tool evaluates the risk of bias across six key domains:

1. Random sequence generation.
2. Allocation concealment.
3. Blinding of participants and personnel.
4. Blinding of outcome assessment.
5. Incomplete outcome data.
6. Selective reporting of outcomes. Each domain was rated as “low risk of bias,” “high risk of bias,” or “unclear risk of bias.” We resolved disagreements through discussion or consultation with a third reviewer.

To assess the risk of bias due to missing results (reporting bias), the availability of published study protocols was verified for each included trial. In the absence of a published protocol, we examined whether all expected primary and secondary outcomes, as described in the methods section of the articles, were reported in the results. We considered studies at low risk of reporting bias if their protocols were publicly available and/or if they reported all expected outcomes. No specific statistical analyses for detecting publication bias (funnel plots) were performed due to the clinical heterogeneity of the interventions and the inability to conduct a meta-analysis. However, the risk of bias assessment for the ‘selective reporting of outcomes’ domain was conducted as part of the Cochrane RoB 2 tool, where we classified all included studies as having a low risk of bias in this domain.

### 2.7 Strategy for data synthesis

For the interpretation of the findings from the included studies, we extracted the effect sizes by calculating Cohen’s d, with statistical significance established at a *p*-value less than 0.05 and 95% confidence intervals (95% CI) used to indicate the precision of the estimates. We interpreted Cohen’s d as follows: values between 0 and 0.2 as a small effect, 0.2–0.5 as moderate, 0.5–0.8 as large, and >0.8 as a very large effect (26). Given that the depression measurement tools used in the clinical trials assigned lower scores to lower levels of depression, a negative Cohen’s d value indicates an improvement in symptoms. The calculations were performed using the Cambridge Effect Size Calculator software (27).

## 3 Results

### 3.1 Study selection

**Figure 1:**
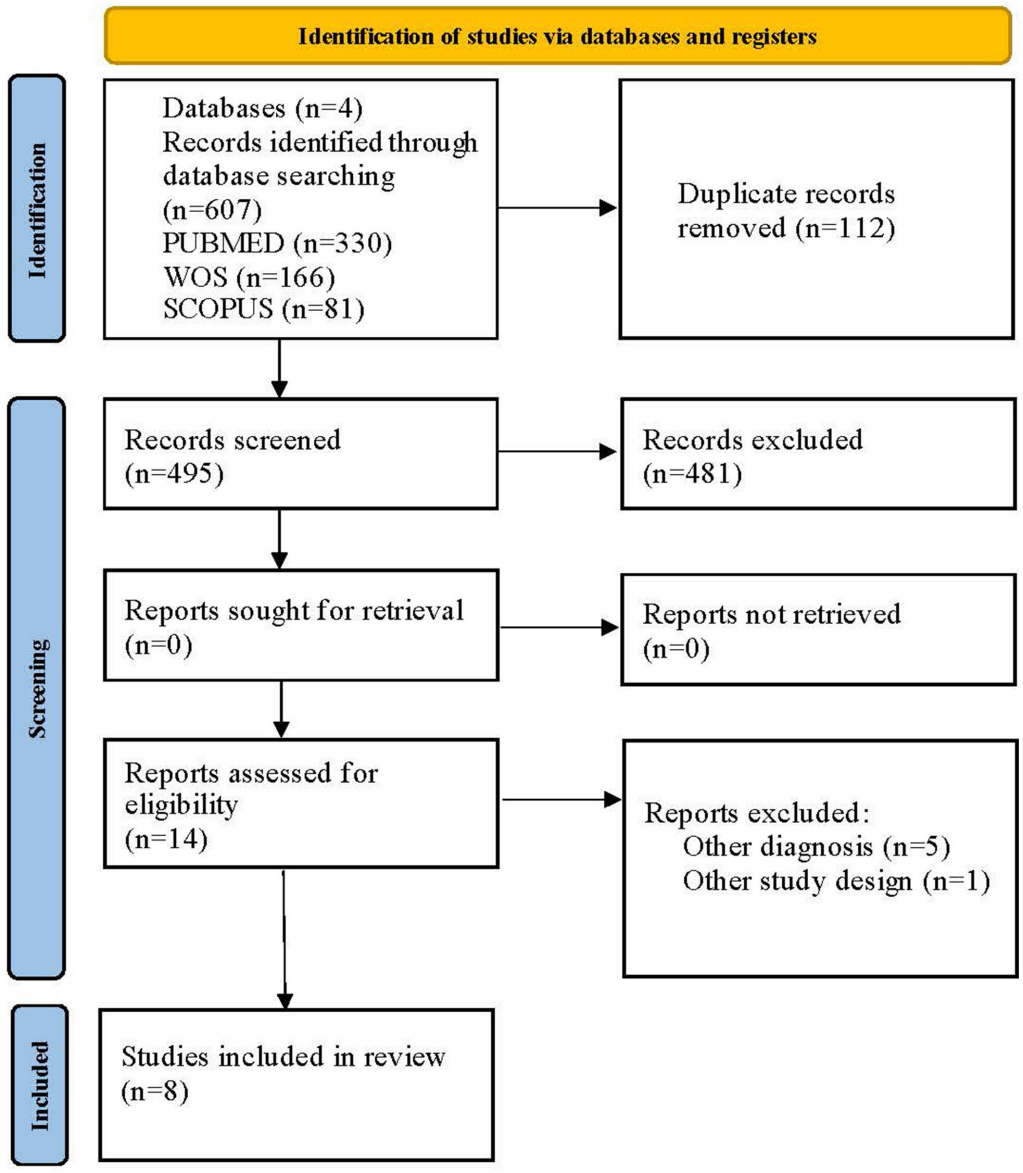
PRISMA 2020 flow diagram. © 2025 by José Lesmes Poveda-López is licensed under Creative Commons Attribution 4.0 International. To view a copy of this license, visit https://creativecommons.org/licenses/by/4.0/.

The initial search across the five databases identified a total of 607 articles. After removing 112 duplicates using the ZOTERO reference management tool, 495 articles underwent title and abstract screening. Following this initial screening, we excluded 481 articles for not meeting the review’s inclusion criteria. The remaining 14 articles were retrieved in full text for a more detailed evaluation. Of these, we excluded six articles after full-text review for the following reasons: not being randomized controlled trials (*n* = 1) and the diagnosis not being exclusively depression (*n* = 5). Finally, eight randomized controlled trials met all eligibility criteria and were included in the systematic review. The study selection process is detailed in the PRISMA flow diagram (Figure 1).

### 3.2 Characteristics of participants and study settings

The eight studies included in the systematic review involved a total of 1,368 participants. The mean age of participants across all studies was over 60 years. Half of the studies were conducted in community settings (*n* = 4), and the other half were carried out in institutions or nursing homes (28–31) (*n* = 4). The majority of participants in most studies were female.

Regarding depression diagnosis, in five studies, the population had a diagnosis of mild to moderate depression, whereas in the remaining three studies, participants presented depressive symptoms without a formal clinical diagnosis, detected using scales. In all studies, participants were permitted to continue their usual pharmacological medication for depression or other comorbidities.

### 3.3 Characteristics of outcome measurement scales

The included studies utilized various measurement scales to assess depressive symptoms validated for the older adult population. The most frequently employed scales were the Geriatric Depression Scale (GDS-15) (32), the Hamilton Depression Rating Scale (HDRS or HAM-D) (33), the Hospital Anxiety and Depression Scale (HADS) (34), and the Center for Epidemiologic Studies Depression Scale (CES-D) (35).

The GDS-15 is a self-administered scale with 15 items that assess the presence of depressive symptoms over the past 2 weeks. A cutoff score of 5/15 is commonly used to indicate the presence of depression, being ideally used due to its simplicity and reliability. The HDRS (or HAM-D) is a hetero-administered scale, consisting of 17, 21, or 24 items, which evaluates a series of depressive symptoms. Higher scores indicate greater severity of depression. It is one of the most widely used scales in clinical research on depression. The HADS is a self-administered scale with 14 items, 7 for anxiety and 7 for depression. It is used to detect symptoms of anxiety and depression in patients with physical illnesses. A cutoff score of 8/21 for the depression subscale suggests the possible presence of a depressive disorder. The CES-D is a 20-item self-administered scale designed to measure depressive symptoms in the general population. Scores above 16 suggest the presence of significant depressive symptoms.

The main characteristics of the subjects and the detailed characteristics of the scales are included in Table 1.

**Table 1:** Characteristics of participants and clinical measures in included studies.

Reference	*N*	Gender (M/F)	Age (Years)	Diagnosis	Measure instrument	Severity of depressive symptoms	Pharmacological treatment	Psychotherapeutic treatment	Patient location
Singh et al. (37)	32; IG: 17, CG: 15	12/20	≥60; 71,3 ± 1,2; IG: 70 ± 1,5, CG: 72 ± 2	Major or minor depression or dysthymia	HDRS IG: 12.3 ± 0.9; CG: 11.4 ± 1.0 GDS-15 IG: 16.9 ± 1.6; CG: 13.9 ± 1.4	Mild symptoms	More than 3 months without prior antidepressant treatment	Not specified	Community-dwelling
Conradsson et al. (29)	191	Not specified	≥65	Depression	GDS-15 4.4 ± 3.2	Mild symptoms	Not specified	Not specified	Institutionalized
Jung et al. (30)	52; IG1: 18, IG2: 17, CG: 17	6/46	≥65	IG1: Depression IG2: Depression plus mild cognitive impairment	Interview	Mild symptoms	Not specified	Not specified	Institutionalized
Aibar-Almazán et al. (38)	110; IG: 55, CG: 55	0/110	≥60; 69.15 ± 8.94	Depression	Hospital Anxiety and Depression Scale (HADS) IG: 5.33 ± 3.85; CG: 5.25 ± 3.54	Mild symptoms	Not specified	Not specified	Community-dwelling
Penninx et al. (36)	439; IG1: 146, IG2: 149; CG: 144	132/307	≥60 68.8 ± 5,6	Major or minor depression	Center for Epidemiologic Studies Depression (CES-D) short scale CG, IG1, IG2>5	Symptom severity varies, 22% major depression	Not specified	Not specified	Community-dwelling
Neviani et al. (28)	121; IG1:37; IG2: 42; CG: 42	35/86	65–85; IG1: 75 ± 6.3; IG2: 75 ± 6.2; CG: 75.6 ± 5.6	Major depression	HDRS IG1: 20.1 ± 3.2; IG2: 19.8 ± 2.6; CG: 20.4 ± 3.4	Severe symptoms	Sertraline	Not specified	Institutionalized
Göksin and Asiret (39)	49; IG: 21; CG: 28	0/49	≥65; 70.24 ± 5.94; IG: 69.19 ± 6.24; CG: 71.03 ± 5.7	Depression	GDS-15 IG: 6.57 ± 4.04; CG: 5.57 ± 4.21	Mild symptoms	Not specified	Not specified	Community-dwelling
Underwood et al. (31)	374; IG: 174; CG:200	278/96	≥65; IG: 86.6 ± 7.4; CG: 86.7 ± 7.8	Depression	GDS-15; IG: 7.4 ± 2.4; CG: 7.6 ± 2.4	Mild symptoms	Antidepressant	Not specified	Institutionalized

IG, Intervention Group; CG, Control Group. Characteristics of participants and clinical measures in included studies © 2025 by José Lesmes Poveda-López is licensed under Creative Commons Attribution 4.0 International. To view a copy of this license, visit https://creativecommons.org/licenses/by/4.0/.

### 3.4 Characteristics of interventions

The physiotherapy interventions applied in the studies were varied, though most focused on therapeutic exercise programs. The main characteristics of these interventions are summarized below and detailed in Table 2.

**Table 2:** Characteristics of physiotherapy interventions in included studies.

Reference	Physiotherapy program	Control condition	Program compliance	Intervention duration	Weekly frequency	Session duration	Other treatments
Singh et al. (37)	Group-based high-intensity resistance training program for large muscle groups (80% 1RM) on supervised gym machines, followed by muscle stretching	Health education program through talks and videos	95%	10 weeks	3/week	50 min	Not specified
Conradsson et al. (29)	Group-based high-intensity functional exercise program	Absence of intervention	Not specified	6 weeks	5/week	Not specified	Not specified
Jung et al. (30)	Full-body physical exercise program and oral muscle exercises. Seated, simple, and repetitive program. Self-administered without supervision	Absence of intervention	100%	6 weeks	2/week	30 min	Music therapy, laughter therapy, and oral health education
Aibar-Almazán et al. (38)	Group-based therapeutic exercise program based on Pilates	Absence of intervention	91,6%	12 weeks	2/week	55 min	Not specified
Penninx et al. (36)	Double group program, IG1: Aerobic walking program 50–70% HRmax plus stretching; IG2: Upper and lower body strength program, 2 sets of 10 reps and progressive	Health education	IG1: 80%; IG2: 82%	18 months	3/week	60 min	Not specified
Neviani et al. (28)	Double group training, IG1: Strength, coordination, breathing, and balance exercise program 70% HRmax; IG2: Progressive interval training program	Absence of intervention	83,5%	24 weeks	3/week	60 min	Not specified
Göksin and Asiret (39)	Individual progressive muscle relaxation exercise program	Absence of intervention	100%	8 weeks	3/week	28 min	Not specified
Underwood et al. (31)	Progressive mixed physical exercise program (aerobic, strength, and balance) increasing in intensity	Health education	70%	12 months	2/week	40–60 min	Not specified

Characteristics of physiotherapy interventions in included studies © 2025 by José Lesmes Poveda-López is licensed under Creative Commons Attribution 4.0 International. To view a copy of this license, visit https://creativecommons.org/licenses/by/4.0/.

#### 3.4.1 Type of intervention

- Therapeutic exercise was the most frequent intervention, present in 6 out of 8 studies. Programs included combinations of aerobic exercise (walking, stationary cycling) (36), strength training (weights, resistance bands) (37), balance, and flexibility (stretching) (28, 31, 37).
- Relaxation Techniques: One study used a combination of exercise and guided relaxation techniques (31).
- Functional Physical Activities: Three studies implemented a program of group functional physical activities adapted to the participants’ capacities (29, 30, 38).

#### 3.4.2 Duration and frequency

The duration of the programs ranged from 8 weeks to 6 months. The most common frequency was 2–3 sessions per week. The duration of each session varied between 30 and 60 min.

#### 3.4.3 Supervision

In most studies, physiotherapists supervised physiotherapy sessions. Some studies included home-based programs with periodic follow-up.

#### 3.4.4 Control group conditions

- Absence of intervention (*n* = 5 studies).
- Placebo or low-intensity non-therapeutic intervention (educational sessions on general health without an active physical component) (*n* = 3 studies).

### 3.5 Description of results

Physical interventions appear to be a promising approach in reducing depressive symptoms, though the variability in study results and methodologies highlights the complexity of this relationship. Specifically, several studies reported significant reductions in depression scale scores such as the GDS-15 (31, 36, 37), the HDRS (28, 30) and the CES-D (39) following programs of exercise, aerobic exercise, a combination of exercise and relaxation, and strength training. While one study on Tai Chi showed a clinically relevant improvement in CES-D, it did not achieve full statistical significance (29). Conversely, a physiotherapy study found no significant differences in HADS scores, though it did observe a trend towards improvement in the depression subscale (38). These findings suggest that a wide range of physical activities can positively impact mental well-being, although the magnitude and consistency of effects may vary depending on the type of intervention and the population studied.

Due to the considerable clinical and methodological heterogeneity among the included studies, a meta-analysis was not feasible. Instead, we conducted a qualitative exploration of potential sources of heterogeneity in the results. This exploration focused on the variability in physiotherapy intervention characteristics (type of technique, duration, or frequency), the measurement scales used to assess depressive symptoms (GDS-15, HDRS, HADS, CES-D, with their differing scoring ranges), and the settings where the studies were conducted (community/home versus residential care facilities).

The most relevant findings from each included study are described below, focusing on the improvement of depressive symptoms (Table 3).

**Table 3:** Description of the results.

Reference	Rating Scale	Initial CG Score	Final CG Score	CG Difference	Initial IG Score	Final IG Score	IG Difference	Difference CG/IG
Singh et al. (37)	HDRS GDS-15	H: 11.4 ± 1.0 GDS-15: 13.9 ± 1.4	H: 8.9 ± 1.3 GDS-15: 12 ± 1.8	*p* > 0.05	H: 12.3 ± 0.9 GDS-15: 16.9 ± 1.6	H: 5.3 ± 1.3 GDS: 8.6 ± 1.8	*p* < 0.05 on both scales	*p* < 0.05 on both scales
Conradsson et al. (29)	GDS-15	3.6 ± 2.9	2.06 ± 2.56	*p* > 0.05	7.8 ± 2.5	6.22 ± 2.53	*p* > 0.05	*p* > 0.05
Jung et al. (30)	Subjective Interview	63.52 ± 10.79	62.52 ± 9.88	*p* > 0.05	61.83 ± 11.24	67.72 ± 9.86	*p* > 0.05	*p* < 0.05
Aibar-Almazán et al. (38)	HADS	5.25 ± 3.54	6.81 ± 3.6	*p* > 0.05	5.33 ± 3.85	3.98 ± 2.93	*p* < 0.05	*p* < 0.05
Penninx et al. (36)	Short CES-D	6.9	5.49	*p* > 0.05	IG1: 6.9 IG2: 6.9	IG1: 4.81 IG2: 4.11	IG1 Vs. IG2 *p* < 0.05	Vs. IG1 *p* > 0.05 Vs. IG2 *p* < 0.05
Neviani et al. (28)	HDRS	20.4 ± 3.4	Not specified	Not specified	IG1: 20.1 ± 3.2 IG2: 19.8 ± 2.6	Not specified	Not specified	Not specified
Göksin and Asiret (39)	GDS-15	5.57 ± 4.21	5.17 ± 4.12	*p* > 0.05	6.57 ± 4.04	3.19 ± 3.37	*p* < 0.05	*p* < 0.05
Underwood et al. (31)	GDS-15	7.6 ± 2.4	4.7 ± 3.2	*p* > 0.05	7.4 ± 2.4	4.5 ± 3.2	*p* > 0.05	*p* > 0.05

IG, Intervention Group; CG, Control Group. Descriptions of the results © 2025 by José Lesmes Poveda-López is licensed under Creative Commons Attribution 4.0 International. To view a copy of this license, visit https://creativecommons.org/licenses/by/4.0/.

**Figure 2:**
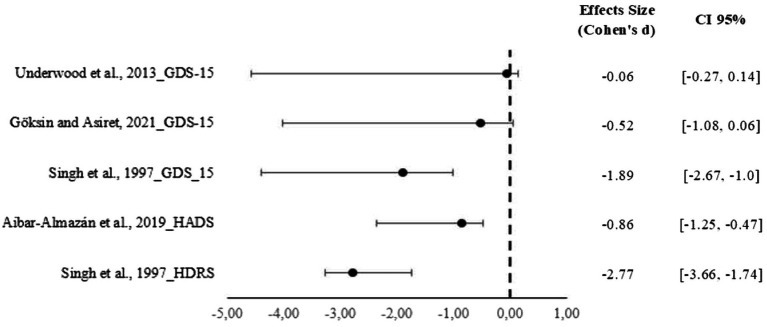
Forest plot of the standardized mean difference (Cohen’s d) for the effect of physiotherapy on depressive symptoms in older adults. © 2025 by Ana Aguilera Gonzalo is licensed under CC BY 4.0. To view a copy of this license, visit https://creativecommons.org/licenses/by/4.0/.

However, the effect size for depression was calculated in the included studies using Cohen’s d, this calculation was feasible for only four (31, 37–39) out of the eight studies included in the systematic review, as the remaining publications did not provide the necessary statistical data for its estimation The analysis revealed varied results, specifically, the findings from Singh et al., on both the HADS scale (−2.77) and the GDS-15 (−1.89), indicated very large effects, suggesting a substantial improvement in depression symptoms within the intervention groups. Aibar-Almazán et al. reported a large effect (−0.86), while Göksin and Asiret showed a moderate effect (−0.52). On the other hand, the study by Underwood et al. presented a trivial effect size (−0.06), indicating a minimal difference between the groups. These findings demonstrate heterogeneity in the magnitude of the interventions’ positive effects (Figure 2).

#### 3.5.1 Results by study setting

The effectiveness of physical interventions appears to vary based on the study setting:

- Community/Population-Based Settings: In studies conducted in community settings or at participants’ homes (36–39), a range of interventions showed positive effects. For instance, studies on aerobic exercise (walking, stationary cycling) (28, 31, 36) and strength training (28, 30, 31, 36–38) consistently reported significant reductions in depression scale scores such as the GDS-15 (37) and the HDRS (38).
- Institutional/Nursing Home Settings: Within institutional settings, interventions also demonstrated a positive impact (28–31). One study on an aerobic walking program showed a clinically relevant improvement in the CES-D (36), even though it did not reach full statistical significance. Additionally, a program of functional physical activities also reported significant reductions in depressive symptoms (29).

#### 3.5.2 Results by intervention type

The range of physical activities varied and their outcomes are described below:

- Therapeutic exercise was the most frequent intervention. Programs that included a combination of aerobic exercise, strength training, and balance and flexibility consistently resulted in significant reductions in depression scores (31). For example, specific studies reported significant improvements in the GDS-15 (31), HDRS (28), and CES-D (36) following these types of programs.
- Relaxation techniques: One study that combined therapeutic exercise with guided relaxation techniques also reported a significant reduction in depressive symptoms (39), suggesting that mind–body practices may enhance the effectiveness of physical exercise.
- Several studies implemented a functional physical activities program which proved to be effective in reducing depressive symptoms (29, 37). These findings indicate that activities adapted to the participants’ capacities, rather than highly structured exercise, can also be a viable approach.

Within the reviewed studies, not all interventions yielded significant results. One physiotherapy study found no significant differences in HADS scores, though it did observe a trend towards improvement in the depression subscale. The findings indicate that the magnitude and consistency of effects may vary depending on the specific type of intervention and the population studied (Table 4).

**Table 4:** Summary table that maps intervention characteristics to outcomes.

Reference	Physiotherapy program	CG difference	IG Difference	Difference CG/IG
Singh et al. (37)	Group-based high-intensity resistance training program for large muscle groups (80% 1RM) on supervised gym machines, followed by muscle stretching	*p* > 0.05	*p* < 0.05	*p* < 0.05
Conradsson et al. (29)	Group-based high-intensity functional exercise program	*p* > 0.05	*p* > 0.05	*p* > 0.05
Jung et al. (30)	Full-body physical exercise program and oral muscle exercises. Seated, simple, and repetitive program. Self-administered without supervision	*p* > 0.05	*p* > 0.05	*p* < 0.05
Aibar-Almazán et al. (38)	Group-based therapeutic exercise program based on Pilates	*p* > 0.05	*p* < 0.05	*p* < 0.05
Penninx et al. (36)	Double group program, IG1: Aerobic walking program 50–70% HRmax plus stretching; IG2: Upper and lower body strength program, 2 sets of 10 reps and progressive	*p* > 0.05	IG1 Vs. IG2 *p* < 0.05	Vs. IG1 *p* > 0.05 Vs. IG2 *p* < 0.05
Neviani et al. (28)	Double group training, IG1: Strength, coordination, breathing, and balance exercise program 70% HRmax; IG2: Progressive interval training program	Not specified	Not specified	Not specified
Göksin and Asiret (39)	Individual progressive muscle relaxation exercise program	*p* > 0.05	*p* < 0.05	*p* < 0.05
Underwood et al. (31)	Progressive mixed physical exercise program (aerobic, strength, and balance) increasing in intensity	*p* > 0.05	*p* > 0.05	*p* > 0.05

IG, Intervention Group; CG, Control Group. Summary table that maps intervention characteristics to outcomes © 2025 by José Lesmes Poveda López is licensed under CC BY 4.0. To view a copy of this license, visit https://creativecommons.org/licenses/by/4.0/.

### 3.6 Assessment of risk of bias in studies

We used in the analysis of the risk of bias of the included randomized controlled trials (RCTs) the Excel tool RoB 2, developed by the Cochrane Library in their Handbook for Systematic Reviews of Interventions Version 5.1.0 (25, 40).

**Figure 3:**
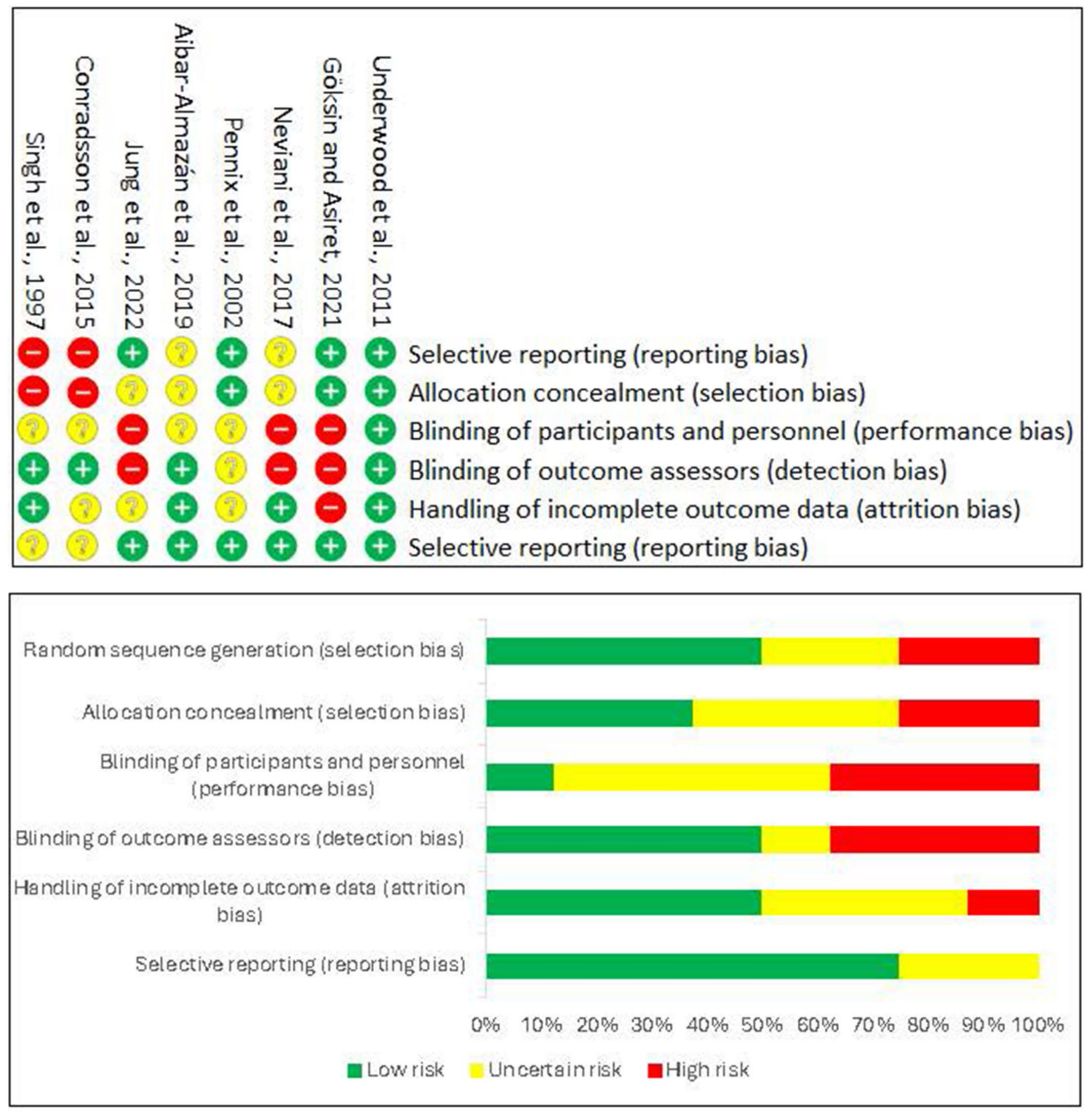
Risk of bias graph. © 2025 by José Lesmes Poveda-López is licensed under Creative Commons Attribution 4.0 International. To view a copy of this license, visit https://creativecommons.org/licenses/by/4.0/.

.The risk of bias assessment, performed using the Cochrane Collaboration’s tool, showed considerable variability among the included studies. Figure 3 presents a visual summary of the risk of bias for each domain and study.

- Random sequence generation: We considered most studies (*n* = 6) at low risk of bias in this domain, as they described adequate randomization methods (computer generation, random number tables). Two studies (*n* = 2) had some concerns due to insufficient description.
- Allocation concealment: Only four studies were at low risk, describing adequate allocation concealment (e.g., sealed opaque envelopes). The remaining four had some concerns, without specifying the method.
- Blinding of participants and personnel: This was a high-risk domain in most studies (*n* = 7), as expected in physiotherapy interventions, due to the difficulty of physical interventions to blind participants and therapists. One study (*n* = 1) was at low risk because it implemented measures to blind outcome assessors, and participants were unaware of their exact assignment. Blinding of outcome assessment: five studies were at low risk, as outcome assessors were blinded to group assignment. Three studies had some concerns, without specifying whether assessors were blinded.
- Incomplete outcome data: Most studies (*n* = 6) were at low risk, with low attrition rates or adequate handling of missing data. Two studies (*n* = 2) had concerns due to a lack of information on attrition.
- Selective reporting of outcomes: We considered all studies (*n* = 8) at low risk in this domain, as protocols were previously published; or all expected primary and secondary outcomes were reported.

Overall, due to the high risk of bias in blinding participants and personnel (an inherent challenge in exercise trials) and some concerns in other domains for a subset of studies, we considered the overall risk of bias for the body of evidence to have some concerns.

### 3.7 Assessment of certainty

The GRADE assessment of the certainty of evidence for the outcome of depression across various studies reveals a consistent pattern in initial ratings and subsequent downgrades. All reviewed studies, being Randomized Controlled Trials (RCTs), commenced with an initial “High quality” rating. However, this initial high quality was systematically compromised by factors leading to a reduction in the overall certainty of the evidence.

A frequently encountered challenge was the risk of bias, often stemming from a lack of explicit details regarding randomization procedures, allocation concealment, and blinding of participants or outcome assessors. This is particularly critical for self-reported outcomes such as depression, where inadequate blinding can influence responses.

Indirectness emerged as another important concern. Researchers frequently conducted the studies in particular populations (postmenopausal Spanish women, nursing home residents, older women, persons with knee osteoarthritis, or those already on sertraline medication) or designed them with precise interventions. These facts introduced indirectness when the review aimed to generalize the findings to broader “older adult” populations or a wider spectrum of “physical exercise.”

Imprecision was a recurrent issue, manifested by small sample sizes or the absence of crucial information such as confidence intervals or *p*-values, which hindered the accurate assessment of observed effect sizes. Finally, the lack of prospective trial registration in many studies indicates a potential for publication bias. Nevertheless, registration in databases such as ClinicalTrials.gov or funding by bodies such as NIHR HTA (Health Technology Assessment) have alleviated these concerns in specific instances.

While RCT provided a strong baseline, we consistently downgraded the certainty of the evidence for depression due to methodological limitations including risk of bias, limited direct applicability to broader populations or interventions (indirectness), imprecision of results, and potential publication bias. Researchers identified no clear factors that would justify increasing the certainty of evidence in any of the analyzed studies. All results are shown in Supplementary File S5 GRADE certainty assessment and Supplementary File S6 GRADE summary.

## 4 Discussion

This systematic review aimed to analyze the scientific evidence regarding the effectiveness of physiotherapy techniques on depressive symptoms in older adult patients. We included eight randomized controlled trials, encompassing 1,368 participants with an average age of over 60 years. Researchers conducted most studies in community settings, which allowed participants to continue their usual antidepressant medication. This reflects a pragmatic approach relevant to real-world clinical practice.

The findings of this review suggest a promising trend towards the improvement of depressive symptoms in older adults through the inclusion of physiotherapy interventions. Most studies (seven out of eight) reported some significant improvement or positive trend in depression scale scores (GDS-15, HDRS, HADS, CES-D) in the intervention group compared to the control group. Therapeutic exercise, in its various modalities such as aerobic (41), strength (42), and balance (43), emerged as the most frequently investigated physiotherapy intervention with the most consistent results. This evidence aligns with the general literature supporting exercise as an effective intervention for mental health, although the specific evidence in older adults with depression remains limited (42, 44).

The results obtained suggest that the incorporation of physiotherapy techniques into the usual treatment plans for older adults with depression can improve their symptoms and would lead to a better quality of life for the affected individuals, while contributing to optimizing healthcare systems (45). Some studies emphasize the important role of physiotherapists in psychiatric units (46, 47), as the benefits of planned therapeutic exercises and muscle relaxation techniques have been proven. Although we failed to identify a physiotherapy program with sufficient validity to demonstrate a reduction in depressive symptoms in older adults, there is clear evidence that physiotherapy (48, 49) may be beneficial. Previous studies not limited to older adults such as Knubben et al. (50) have shown positive effects of physiotherapy on depression through strength exercises and, like Schimitter et al. (51), through aerobic exercise programs.

Studies by Gildengers et al. (52), Eumura et al. (53), and Cangöz et al. (54) demonstrated that a programmed physiotherapy intervention helps to improve depressive symptoms in patients who also have cognitive deficits, with both measures being significantly improved. Physiotherapy also improves depressive symptoms associated with other pathologies, such as obesity related to cancer (55), chronic pain (56) or diabetes (3).

However, it is crucial to interpret these results cautiously due to several limitations. As described above, researchers systematically assessed the certainty of evidence for the effectiveness of physiotherapy interventions on depressive symptoms in older adult patients, using the GRADE framework. This rigorous analysis consistently revealed that researchers rated the overall quality of evidence for almost all included studies as either low or very low. This classification is primarily due to significant methodological concerns, including high risk of bias in multiple domains (lack of blinding of participants and personnel, high attrition rates), substantial heterogeneity of intervention protocols and outcome measures, and sometimes imprecision due to small sample sizes. Consequently, these findings suggest that while there is a promising trend, further high-quality research is critically needed to increase confidence in the reported effects and to establish definitive recommendations for clinical practice. One of the main limitations is the heterogeneity of physiotherapy interventions, as highlighted by Hidalgo et al. (19), who indicated that previous studies on the effectiveness of physiotherapy techniques, especially therapeutic exercises, have been highly heterogeneous, preventing a solid conclusion and highlighting the need for further research. Although the focus was on exercise, intensities, frequencies, and durations varied considerably across the studies. This limited the ability to perform a meta-analysis and obtain an aggregate effect estimate, which made it difficult to determine optimal treatment protocols.

Programs that have led to greater improvement in depression rates are those conducted by recruiting the population from the community, compared to those conducted in nursing homes and care facilities for older adults. This aligns with the findings of Blumenthal et al. (57) regarding home-supervised interventions, which showed better outcomes in terms of strength and a lower dropout rate, addressing one of the biggest challenges in this population. However, studies such as that by Pitkala et al. (58), which also link these programs to dementia-related issues, do not definitively clarify whether this is an equally central factor in the change of symptomatology.

Another important limitation was the risk of bias, particularly concerning the blinding of participants and personnel. In physical interventions, it is inherently difficult to achieve complete blinding, which may have introduced performance or detection bias in some studies. In most low-risk studies, researchers blinded the outcome subjects, but the participants’ perception of the treatment could influence the reported results. This fact aligns with several reviews on similar topics, such as Sun et al. (59), who evaluated the effect of physical exercise on cognitive symptoms in older adults, or Heissel et al. (60), who assessed various non-pharmacological procedures on depressive symptoms in the general population.

The heterogeneity of the populations and interventions in the included studies emerged as a significant concern, directly impacting on the generalizability of our findings. This indirectness in the evidence resulted from the fact that the researchers frequently conducted studies on very specific populations, such as postmenopausal Spanish women, nursing home residents, or individuals with knee osteoarthritis (26–28, 32, 39). While these targeted studies provide valuable insights for their respective cohorts, the lack of uniformity across the PICO elements prevents a direct and robust comparison, highlighting the need for more standardized research in this field. Consequently, it remains a challenge to draw clear conclusions that are generally applicable to the entire heterogeneous population of older adults. Future research should focus on diverse and representative samples to enhance the external validity of the results and provide better clinical guidelines for the broader population of older adults with depression.

Furthermore, the variability of the depression scales used, although all validated, adds a layer of complexity to the direct comparison of results. Pérez Bedoya et al. (44) and Zhang et al. (61) also indicate that this is one of the main problems when comparing studies, in which the variable sensitivity and specificity among these tools could have led to different effects. This variability is a source of heterogeneity that complicates the interpretation of findings, as highlighted by Li et al. (62) in their review on mind–body exercise, stating that ‘differences in experimental design, time, frequency, duration, and outcome measurement method would lead to different results, causing difficulty in explanation.’ The distinction between a formal diagnosis of depression and the presence of depressive symptoms as determined by a scale should also be considered. However, the inclusion of both types of populations increases the generalizability of findings to a broader range of older adults with mental health issues.

Despite the limitations of conducting a meta-analysis due to heterogeneity and lack of available data (50, 63), our analysis of effect sizes (Cohen’s d) in the relevant studies revealed a consistent trend (64). Although the magnitude of the effects varied, all findings indicated an improvement in depressive symptoms within the intervention groups, given the context of the depression scales used. This variability in magnitude reinforces the conclusion of high heterogeneity in the studies, but the consistency in the direction of the effect provides promising evidence that physiotherapy interventions are beneficial for depression in older adults (58, 63).

Despite these limitations, the strengths of this review include a rigorous methodology following PRISMA guidelines, an exhaustive search across multiple databases to minimize publication bias, and the exclusive inclusion of randomized controlled trials, which represent the highest level of evidence. The clinical relevance of including studies from community settings and with participants continuing their usual medication underscores the applicability of the findings in real-world practice.

Physiotherapy, especially therapeutic exercise, emerges as a promising and safe addition to conventional treatments of depression in older adults. Given the high prevalence of depression in this population and the limitations of current treatments, physiotherapy offers a non-pharmacological alternative with potential benefits extending beyond mental health, including improved physical function and overall quality of life. More high-quality research, specifically well-designed randomized controlled trials, is urgently needed. Future studies should focus on standardizing physiotherapy intervention protocols (type, intensity, duration, frequency) to enable meta-analyses and determine the optimal “dose”; including active comparison groups and appropriate placebos to better control for attention and expectation effects; increasing sample size to enhance statistical power; conducting long-term follow-up to evaluate the sustainability of effects; and exploring the underlying mechanisms (e.g., neurobiological changes, psychosocial factors) by which physiotherapy improves depressive symptoms in older adults.

## 5 Conclusion

Findings of this systematic review suggest a positive trend in the effectiveness of physiotherapy techniques on depressive symptoms in older adults, the current evidence is limited and notably heterogeneous. Therefore, we urgently require further rigorous randomized controlled trials. These future studies should aim to standardize physiotherapy intervention protocols (including type, intensity, duration, and frequency) to confirm efficacy, enable robust meta-analyses, and ultimately establish evidence-based clinical guidelines for this vulnerable population.

## Data Availability

The original contributions presented in the study are included in the article/Supplementary material, further inquiries can be directed to the corresponding author.
