# Supplementary material for: Effectiveness of physiotherapy techniques on depressive symptoms in older adults: a systematic review
Source: Front Public Health. 2025 Sep 17;13:1671788. doi: 10.3389/fpubh.2025.1671788 (PMC12483849; doi:10.3389/fpubh.2025.1671788)
Supplement: Supplementary file 1 [file Supplementary_file_1.docx]

Supplementary Material

# Supplementary Material

File S1. PRISMA_2020_checklist

File S2. Eligibility Criteria based on PICOS Framework

File S3. Search terms

File S4. Search strategy

File S5. GRADE Certainty assessment

File S1

| **Section and Topic** | **Item #** | **Checklist item** | **Location where item is reported** |
| --- | --- | --- | --- |
| **TITLE** | | |  |
| Title | 1 | Identify the report as a systematic review. | Pag. 1 |
| **ABSTRACT** | | |  |
| Abstract | 2 | See the PRISMA 2020 for Abstracts checklist. | Pag. 2 |
| **INTRODUCTION** | | |  |
| Rationale | 3 | Describe the rationale for the review in the context of existing knowledge. | Pag. 3 |
| Objectives | 4 | Provide an explicit statement of the objective(s) or question(s) the review addresses. | Pag. 4 |
| **METHODS** | | |  |
| Eligibility criteria | 5 | Specify the inclusion and exclusion criteria for the review and how studies were grouped for the syntheses. | Pag. 4 |
| Information sources | 6 | Specify all databases, registers, websites, organisations, reference lists and other sources searched or consulted to identify studies. Specify the date when each source was last searched or consulted. | Pag. 4 |
| Search strategy | 7 | Present the full search strategies for all databases, registers and websites, including any filters and limits used. | Pag. 4 |
| Selection process | 8 | Specify the methods used to decide whether a study met the inclusion criteria of the review, including how many reviewers screened each record and each report retrieved, whether they worked independently, and if applicable, details of automation tools used in the process. | Pag. 5 |
| Data collection process | 9 | Specify the methods used to collect data from reports, including how many reviewers collected data from each report, whether they worked independently, any processes for obtaining or confirming data from study investigators, and if applicable, details of automation tools used in the process. | Pag. 5 |
| Data items | 10a | List and define all outcomes for which data were sought. Specify whether all results that were compatible with each outcome domain in each study were sought (e.g. for all measures, time points, analyses), and if not, the methods used to decide which results to collect. | Pag. 5 |
|  | 10b | List and define all other variables for which data were sought (e.g. participant and intervention characteristics, funding sources). Describe any assumptions made about any missing or unclear information. | Pag. 5 |
| Study risk of bias assessment | 11 | Specify the methods used to assess risk of bias in the included studies, including details of the tool(s) used, how many reviewers assessed each study and whether they worked independently, and if applicable, details of automation tools used in the process. | Pag. 5 |
| Effect measures | 12 | Specify for each outcome the effect measure(s) (e.g. risk ratio, mean difference) used in the synthesis or presentation of results. | N/A |
| Synthesis methods | 13a | Describe the processes used to decide which studies were eligible for each synthesis (e.g. tabulating the study intervention characteristics and comparing against the planned groups for each synthesis (item #5)). | N/A |
|  | 13b | Describe any methods required to prepare the data for presentation or synthesis, such as handling of missing summary statistics, or data conversions. | N/A |
|  | 13c | Describe any methods used to tabulate or visually display results of individual studies and syntheses. | Pag. 5 |
|  | 13d | Describe any methods used to synthesize results and provide a rationale for the choice(s). If meta-analysis was performed, describe the model(s), method(s) to identify the presence and extent of statistical heterogeneity, and software package(s) used. | Pag. 6 |
|  | 13e | Describe any methods used to explore possible causes of heterogeneity among study results (e.g. subgroup analysis, meta-regression). | Pag. 6 |
|  | 13f | Describe any sensitivity analyses conducted to assess robustness of the synthesized results. | N/A |
| Reporting bias assessment | 14 | Describe any methods used to assess risk of bias due to missing results in a synthesis (arising from reporting biases). | Pag. 6 |
| Certainty assessment | 15 | Describe any methods used to assess certainty (or confidence) in the body of evidence for an outcome. | Pag. 6 |
| **RESULTS** | | |  |
| Study selection | 16a | Describe the results of the search and selection process, from the number of records identified in the search to the number of studies included in the review, ideally using a flow diagram. | Pag. 7 |
|  | 16b | Cite studies that might appear to meet the inclusion criteria, but which were excluded, and explain why they were excluded. | Pag. 7 |
| Study characteristics | 17 | Cite each included study and present its characteristics. | Pag. 8 |
| Risk of bias in studies | 18 | Present assessments of risk of bias for each included study. | Pag. 10 |
| Results of individual studies | 19 | For all outcomes, present, for each study: (a) summary statistics for each group (where appropriate) and (b) an effect estimate and its precision (e.g. confidence/credible interval), ideally using structured tables or plots. | Pag. 9-10 |
| Results of syntheses | 20a | For each synthesis, briefly summarise the characteristics and risk of bias among contributing studies. | Pag. 7/9 |
|  | 20b | Present results of all statistical syntheses conducted. If meta-analysis was done, present for each the summary estimate and its precision (e.g. confidence/credible interval) and measures of statistical heterogeneity. If comparing groups, describe the direction of the effect. | N/A |
|  | 20c | Present results of all investigations of possible causes of heterogeneity among study results. | Pag. 9 |
|  | 20d | Present results of all sensitivity analyses conducted to assess the robustness of the synthesized results. | N/A |
| Reporting biases | 21 | Present assessments of risk of bias due to missing results (arising from reporting biases) for each synthesis assessed. | Pag. 10 |
| Certainty of evidence | 22 | Present assessments of certainty (or confidence) in the body of evidence for each outcome assessed. | Pag. 11 |
| **DISCUSSION** | | |  |
| Discussion | 23a | Provide a general interpretation of the results in the context of other evidence. | Pag. 12 |
|  | 23b | Discuss any limitations of the evidence included in the review. | Pag. 12-13 |
|  | 23c | Discuss any limitations of the review processes used. | Pag. 13 |
|  | 23d | Discuss implications of the results for practice, policy, and future research. | Pag. 14 |
| **OTHER INFORMATION** | | |  |
| Registration and protocol | 24a | Provide registration information for the review, including register name and registration number, or state that the review was not registered. | Pag. 4 |
|  | 24b | Indicate where the review protocol can be accessed, or state that a protocol was not prepared. | Pag. 4 |
|  | 24c | Describe and explain any amendments to information provided at registration or in the protocol. | N/A |
| Support | 25 | Describe sources of financial or non-financial support for the review, and the role of the funders or sponsors in the review. | Pag. 15 |
| Competing interests | 26 | Declare any competing interests of review authors. | Pag. 15 |
| Availability of data, code and other materials | 27 | Report which of the following are publicly available and where they can be found: template data collection forms; data extracted from included studies; data used for all analyses; analytic code; any other materials used in the review. | Pag. 15 |

File S2

| **Item** | **Inclusion Criteria** | **Exclusion Criteria** |
| --- | --- | --- |
| **P (Population/Participants)** | Patients aged over 60 years with a medical diagnosis of depression or depressive symptoms detected by a recognized depression scale. Studies where the sample is exclusively elderly or where the mean age of the sample is over 60 years are included. | Patients with other mental disorders (psychosis, bipolar disorder, schizophrenia, etc.). Patients with severe cognitive impairment or advanced dementias that prevent participation in interventions. Patients with severe or terminal illnesses that severely limit their participation. |
| **I (Intervention)** | Intervention program with physiotherapy techniques with a minimum duration of 6 weeks and complementary to the usual treatment of depression | Interventions unrelated to physiotherapy (e.g., medication exclusively, psychotherapy without a physical component, acupuncture, occupational therapy). |
| **C (Comparison)** | Control group receiving conventional treatment for depression (pharmacological, psychotherapeutic) or placebo/no additional intervention. | Studies without a control or comparison group. |
| **O (Outcomes)** | Measurement of depressive symptoms using validated depression scales. | Studies that do not measure the effect of interventions on depressive symptoms. |
| **S (Study Design)** | Randomized Controlled Trials (RCTs). | Narrative reviews, meta-analyses, observational studies, qualitative studies, case series, case reports, editorials, letters to the editor, book chapters. |

File S3

| - **Concept** | - **Terms searched** | |
| --- | --- | --- |
|  | - **MESH** | - **Free-text words** |
| - Physiotherapy | - Physical therapy modalities | - Modalities, Physical Therapy - Physiotherapy (Techniques) - Physiotherapy Techniques - Physical Therapy - Physical therapies - Therapy, physical |
| - Aged, Frail | - Aged - Aged, 80 and over - Octogenarians - Nonagenarians - Frail elderly | - Elderly - Oldest old - Elderly, frail - Frail elder - Frail older adults - Adult, frail older |
| - Depression | - Depression - Depressive disorder | - Depressive symptoms - Depressive symptom - Symptom, depressive - Emotional depression - Depression, emotional - Disorder, depressive - Disorders, depressive - Depressive syndrome - Depressive syndromes |
| - Measuring mental state instruments | - Patient health questionnaire - Psychiatric status rating scales | - Yesavage Scale - GDS scale - Hamilton Scale |

File S4

| **Database** | **Search Strategy** |
| --- | --- |
| **PubMed** | (“physical therapy modalities”[Mesh] OR “Modalities, Physical Therapy” OR “Physiotherapy (Techniques)” OR “Physiotherapy Techniques” OR “Physical Therapy” OR “Physical therapies” OR “Therapy, physical” OR “physiotherapy”) AND (“aged”[Mesh] OR “aged, 80 and over”[Mesh] OR “octogenarians”[Mesh] OR “Nonagenarians”[Mesh] OR “frail elderly”[Mesh] OR Elderly OR “Oldest old” OR “Elderly, frail” OR “Frail elder” OR “Frail older adults” OR “Adult, frail older”) AND (“depression”[Mesh] OR “depressive disorder, major”[Mesh] OR “depressive disorder”[Mesh] OR “Depressive symptoms” OR “Depressive symptom” OR “Symptom, depressive” OR “Emotional depression” OR “Depression, emotional” OR “Disorder, depressive” OR “Disorders, depressive” OR “Depressive syndrome” OR “Depressive syndromes”) AND (“Patient health questionnaire”[Mesh] OR “Psychiatric status rating scales”[Mesh] OR “GDS scale” OR “Yesavage scale” OR “Hamilton scale”) |
| **Web of Science (WOS)** | (“physical therapy modalities” OR “Modalities, Physical Therapy” OR “Physiotherapy (Techniques)” OR “Physiotherapy Techniques” OR “Physical Therapy” OR “Physical therapies” OR “Therapy, physical”) AND (“aged” OR “aged, 80 and over” OR “octogenarians” OR “Nonagenarians” OR “frail elderly” OR Elderly OR “Oldest old” OR “Elderly, frail” OR “Frail elder” OR “Frail older adults” OR “Adult, frail older”) AND (“depression” OR “depressive disorder, major” OR “depressive disorder” OR “Depressive symptoms” OR “Depressive symptom” OR “Symptom, depressive” OR “Emotional depression” OR “Depression, emotional” OR “Disorder, depressive” OR “Disorders, depressive” OR “Depressive syndrome” OR “Depressive syndromes”) AND (“Patient health questionnaire” OR “Psychiatric status rating scales” OR “GDS scale” OR “Yesavage scale” OR “Hamilton Scale”) |
| **Scopus** | (“physical therapy modalities” OR “Modalities, Physical Therapy” OR “Physiotherapy (Techniques)” OR “Physiotherapy Techniques” OR “Physical Therapy” OR “Physical therapies” OR “Therapy, physical”) AND (“aged” OR “aged, 80 and over” OR “octogenarians” OR “Nonagenarians” OR “frail elderly” OR Elderly OR “Oldest old” OR “Elderly, frail” OR “Frail elder” OR “Frail older adults” OR “Adult, frail older”) AND (“depression” OR “depressive disorder, major” OR “depressive disorder” OR “Depressive symptoms” OR “Depressive symptom” OR “Symptom, depressive” OR “Emotional depression” OR “Depression, emotional” OR “Disorder, depressive” OR “Disorders, depressive” OR “Depressive syndrome” OR “Depressive syndromes”) AND (“Patient health questionnaire” OR “Psychiatric status rating scales” OR “GDS scale” OR “Yesavage scale” OR “Hamilton Scale”) |
| **Cochrane Library** | (“physical therapy modalities” OR “Modalities, Physical Therapy” OR “Physiotherapy (Techniques)” OR “Physiotherapy Techniques” OR “Physical Therapy” OR “Physical therapies” OR “Therapy, physical”) AND (“aged” OR “aged, 80 and over” OR “octogenarians” OR “Nonagenarians” OR “frail elderly” OR Elderly OR “Oldest old” OR “Elderly, frail” OR “Frail elder” OR “Frail older adults” OR “Adult, frail older”) AND (“depression” OR “depressive disorder, major” OR “depressive disorder” OR “Depressive symptoms” OR “Depressive symptom” OR “Symptom, depressive” OR “Emotional depression” OR “Depression, emotional” OR “Disorder, depressive” OR “Disorders, depressive” OR “Depressive syndrome” OR “Depressive syndromes”) AND (“Patient health questionnaire” OR “Psychiatric status rating scales” OR “GDS scale” OR “Yesavage scale” OR “Hamilton Scale”) |

File S5

| **GRADE criteria** | **Rating**  (circle one) | **Footnotes**  (explain reasons for down- or upgrading) | **Quality of the evidence** (Circle one) |
| --- | --- | --- | --- |
| **Outcome: Depression**  **Aibar et al. (2019)** | | | |
| **Study design** | RCT (starts as high quality)  Non-RCT (starts as low quality) |  |   High    Moderate    Low    Very Low |
| **Risk of Bias**  *(use the Cochrane Risk of Bias tables and figures)* | No  serious (-1)  very serious (-2) | Randomization and concealment: Registered on ClinicalTrials.gov (NCT03201107) suggests good methodology, but snippet doesn't detail allocation concealment.  Blinding: Not blinded for participants/personnel (normal for exercise); blinding of outcome assessors (important for self-reported outcomes like depression/anxiety) not mentioned.  Incomplete data: Not assessable from snippet. |  |
| **Inconsistency** | No  serious (-1)  very serious (-2) |  |  |
| **Indirectness** | No  serious (-1)  very serious (-2) | Population: Spanish postmenopausal women aged 60+. If review is for "older adults" in general, this introduces indirectness.  Intervention: Specific Pilates training. May be indirect if review is about broader "physical exercise".  Outcomes: Direct (depression, anxiety). |  |
| **Imprecision** | No  serious (-1)  very serious (-2) | N=110, significant p-values and effect sizes (though not provided in snippet, inferred from language). Study appears to be adequately powered. |  |
| **Publication Bias** | Undetected  Strongly suspected (-1) | Prospective registration on ClinicalTrials.gov (NCT03201107) mitigates publication bias. |  |
| **Other**  (upgrading factors, circle all that apply) | Large effect (+1 or +2)  Dose response (+1 or +2) No Plausible confounding (+1 or +2) | No clear reasons to upgrade certainty. |  |

| **GRADE criteria** | **Rating**  (circle one) | **Footnotes**  (explain reasons for down- or upgrading) | **Quality of the evidence** (Circle one) |
| --- | --- | --- | --- |
| **Outcome: Depression**  **Conradsson et al. (2009)** | | | |
| **Study design** | RCT (starts as high quality)  Non-RCT (starts as low quality) |  |   High    Moderate    Low    Very Low |
| **Risk of Bias**  *(use the Cochrane Risk of Bias tables and figures)* | No  serious (-1)  very serious (-2) | Randomization and concealment: Cluster-randomized (care homes), but specific details of sequence generation and concealment not detailed in snippet.  Blinding: Blinding not mentioned for depressive symptoms outcomes (self-reports).  Other biases: Risk of contamination in cluster design if not adequately addressed in analysis. |  |
| **Inconsistency** | No  serious (-1)  very serious (-2) | Not informed |  |
| **Indirectness** | No  serious (-1)  very serious (-2) | Population: Older people living in residential care facilities. This is a very specific and frail population. If review is for "older adults" in the community, this introduces indirectness.  Intervention: High-intensity functional exercise program.  Comparator: Not specified in snippet.  Outcomes: Depressive symptoms and psychological well-being (direct). |  |
| **Imprecision** | No  serious (-1)  very serious (-2) | Not assessable |  |
| **Publication Bias** | Undetected  Strongly suspected (-1) | No mention of prospective trial registration. |  |
| **Other**  (upgrading factors, circle all that apply) | Large effect (+1 or +2)  Dose response (+1 or +2) No Plausible confounding (+1 or +2) | No clear reasons to upgrade certainty. |  |

| **GRADE criteria** | **Rating**  (circle one) | **Footnotes**  (explain reasons for down- or upgrading) | **Quality of the evidence** (Circle one) |
| --- | --- | --- | --- |
| **Outcome: Depression**  **Göksin et al. (2021)** | | | |
| **Study design** | RCT (starts as high quality)  Non-RCT (starts as low quality) |  |   High    Moderate    Low    Very Low |
| **Risk of Bias**  *(use the Cochrane Risk of Bias tables and figures)* | No  serious (-1)  very serious (-2) | Randomization and concealment: "Simple random sampling" used, but no details on sequence generation or concealment.  Blinding: Blinding not mentioned (critical for self-reported outcomes like depression).  Incomplete data: Attrition rates not mentioned.  Other biases: Very small sample size. |  |
| **Inconsistency** | No  serious (-1)  very serious (-2) | Not informed |  |
| **Indirectness** | No  serious (-1)  very serious (-2) | Population: Specifically "elderly women" (not older adults in general).  Intervention: Progressive Muscle Relaxation (direct).  Comparator: Not specified ("controls").  This may be indirect if a specific comparator is required.  Outcomes: Direct (depression, adaptation to old age). |  |
| **Imprecision** | No  serious (-1)  very serious (-2) | Sample Size: Very small (N=49 total; 21 intervention, 28 control). This leads to a high probability of imprecision. Confidence Intervals: Not provided in snippet. |  |
| **Publication Bias** | Undetected  Strongly suspected (-1) | No mention of prospective trial registration. |  |
| **Other**  (upgrading factors, circle all that apply) | Large effect (+1 or +2)  Dose response (+1 or +2) No Plausible confounding (+1 or +2) | No clear reasons to upgrade certainty. |  |

| **GRADE criteria** | **Rating**  (circle one) | **Footnotes**  (explain reasons for down- or upgrading) | **Quality of the evidence** (Circle one) |
| --- | --- | --- | --- |
| **Outcome: Depression**  **Jung et al. (2022)** | | | |
| **Study design** | RCT (starts as high quality)  Non-RCT (starts as low quality) |  |   High    Moderate    Low    Very Low |
| **Risk of Bias**  *(use the Cochrane Risk of Bias tables and figures)* | No  serious (-1)  very serious (-2) | Randomization and concealment: Specific methods for sequence generation or concealment not detailed.  Blinding: Blinding not mentioned for mental health outcomes (self-reports).  Incomplete data: Attrition rate and handling not mentioned. |  |
| **Inconsistency** | No  serious (-1)  very serious (-2) | Not informed |  |
| **Indirectness** | No  serious (-1)  very serious (-2) | Population: Older people.  Intervention: "Integrative Cognitive Function Improvement Program." This is very broad and may not be direct if review seeks effects of specific "physical exercise".  Outcomes: Mental health (direct, if depression included), cognitive function, and oral health. |  |
| **Imprecision** | No  serious (-1)  very serious (-2) | Snippet does not provide sample size, CIs, or p-values to assess precision. Requires full text. |  |
| **Publication Bias** | Undetected  Strongly suspected (-1) | No mention of prospective trial registration. |  |
| **Other**  (upgrading factors, circle all that apply) | Large effect (+1 or +2)  Dose response (+1 or +2) No Plausible confounding (+1 or +2) | No clear reasons to upgrade certainty. |  |

| **GRADE criteria** | **Rating**  (circle one) | **Footnotes**  (explain reasons for down- or upgrading) | **Quality of the evidence** (Circle one) |
| --- | --- | --- | --- |
| **Outcome: Depression**  **Neviani et al. (2017)** | | | |
| **Study design** | RCT (starts as high quality)  Non-RCT (starts as low quality) |  |   High    Moderate    Low    Very Low |
| **Risk of Bias**  *(use the Cochrane Risk of Bias tables and figures)* | No  serious (-1)  very serious (-2) | Randomization and concealment: Specific methods not detailed. Blinding: "Single-blinded" (likely assessors, but not participants/personnel). Incomplete data: Attrition rate and handling not mentioned. |  |
| **Inconsistency** | No  serious (-1)  very serious (-2) | Differential effects between progressive aerobic exercise (significant) and non-progressive (non-significant) compared to control. This is internal inconsistency if generalizing about "exercise". |  |
| **Indirectness** | No  serious (-1)  very serious (-2) | Intervention: Exercise as "add-on" to sertraline (not standalone).  Population: Late-life depressed patients already on sertraline and without severe cognitive impairment. High indirectness if review seeks exercise as primary treatment or in a medication-broader population.  Comparator: Sertraline alone.  Outcomes: Direct (cognition, disability, implicit depression). |  |
| **Imprecision** | No  serious (-1)  very serious (-2) | Sample size of 121 (moderate). P-values and effect sizes provided (for MoCA, disability). Non-significant findings for S+NPE could be imprecision or a true null effect. |  |
| **Publication Bias** | Undetected  Strongly suspected (-1) | No mention of prospective trial registration. |  |
| **Other**  (upgrading factors, circle all that apply) | Large effect (+1 or +2)  Dose response (+1 or +2) No Plausible confounding (+1 or +2) | No clear reasons to upgrade certainty. |  |

| **GRADE criteria** | **Rating**  (circle one) | **Footnotes**  (explain reasons for down- or upgrading) | **Quality of the evidence** (Circle one) |
| --- | --- | --- | --- |
| **Outcome: Depression**  **Pennix et al. (2002)** | | | |
| **Study design** | RCT (starts as high quality)  Non-RCT (starts as low quality) |  |   High    Moderate    Low    Very Low |
| **Risk of Bias**  *(use the Cochrane Risk of Bias tables and figures)* | No  serious (-1)  very serious (-2) | Randomization and concealment: Incomplete details on randomization/concealment.  Blinding: Blinding of outcome assessors not explicitly mentioned (important for self-reported depression).  Incomplete data: Potential for high attrition over 18 months, impact unclear if not handled robustly (ITT). |  |
| **Inconsistency** | No  serious (-1)  very serious (-2) | Snippet does not contain enough results to evaluate internal consistency across different outcomes or consistency with other studies. |  |
| **Indirectness** | No  serious (-1)  very serious (-2) | Population: Older people (60+) with knee osteoarthritis and high/low depressive symptomatology. This is a specific population; if PICO is for general older adults or those with depression without OA, it introduces indirectness.  Intervention/Comparator/Outcomes: Seem direct if PICO matches. |  |
| **Imprecision** | No  serious (-1)  very serious (-2) | Large initial sample size (N=439) suggests adequate power. Reports "significantly lowered depressive symptoms." Without full CIs, exact precision cannot be confirmed, but initial impression is generally good. |  |
| **Publication Bias** | Undetected  Strongly suspected (-1) | No mention of prospective trial registration. |  |
| **Other**  (upgrading factors, circle all that apply) | Large effect (+1 or +2)  Dose response (+1 or +2) No Plausible confounding (+1 or +2) | No clear reasons to upgrade certainty. |  |

| **GRADE criteria** | **Rating**  (circle one) | **Footnotes**  (explain reasons for down- or upgrading) | **Quality of the evidence** (Circle one) |
| --- | --- | --- | --- |
| **Outcome: Depression**  **Singh et al. (1997)** | | | |
| **Study design** | RCT (starts as high quality)  Non-RCT (starts as low quality) |  |   High    Moderate    Low    Very Low |
| **Risk of Bias**  *(use the Cochrane Risk of Bias tables and figures)* | No  serious (-1)  very serious (-2) | Randomization and concealment: Specific methods for sequence generation or concealment not detailed.  Blinding: Participants not blinded (exercise intervention); blinding of outcome assessors for depression outcomes (self-reports) not mentioned.  Incomplete data: 32 participants "completed the study" suggests possible exclusion of non-completers (not ITT).  Other biases: Small sample size. |  |
| **Inconsistency** | No  serious (-1)  very serious (-2) | Snippet doesn't allow assessment of consistency with other studies. Internally, a significant reduction in all depression measures is indicated. |  |
| **Indirectness** | No  serious (-1)  very serious (-2) | Population: Depressed elders (HAM-D ≥ 18), with major/minor depression or dysthymia. If review is for general older adult population, could be indirect.  Intervention: Supervised progressive resistance training (3 times/week). Seems direct if PICO is specific.  Comparator: Attention-control group.  Outcomes: Direct (depression). |  |
| **Imprecision** | No  serious (-1)  very serious (-2) | Sample Size: Very small (N=32). This increases uncertainty of results, even if "significant reductions" are reported. Effects may be overestimated.  Confidence Intervals: Not provided. |  |
| **Publication Bias** | Undetected  Strongly suspected (-1) | No mention of prospective trial registration. |  |
| **Other**  (upgrading factors, circle all that apply) | Large effect (+1 or +2)  Dose response (+1 or +2) No Plausible confounding (+1 or +2) | No clear reasons to upgrade certainty. |  |

| **GRADE criteria** | **Rating**  (circle one) | **Footnotes**  (explain reasons for down- or upgrading) | **Quality of the evidence** (Circle one) |
| --- | --- | --- | --- |
| **Outcome: Depression**  **Underwood et al. 2013** | | | |
| **Study design** | RCT (starts as high quality)  Non-RCT (starts as low quality) |  |   High    Moderate    Low    Very Low |
| **Risk of Bias**  *(use the Cochrane Risk of Bias tables and figures)* | No  serious (-1)  very serious (-2) | Randomization and concealment: Cluster design, but explicit details not in snippet.  Blinding: Assessors blinded, but not participants/personnel (normal for exercise).  Other biases: Risk of contamination inherent in cluster design in care homes. |  |
| **Inconsistency** | No  serious (-1)  very serious (-2) | Full text needed to assess heterogeneity of results with other studies or internally (though "no significant" is mentioned). |  |
| **Indirectness** | No  serious (-1)  very serious (-2) | Population: Care home residents (very specific and frail). Indirect if review is for general older adults.  Intervention: "Exercise intervention" (general description). Could be indirect if seeking a very specific exercise type.  Comparator: "Usual care" (can vary).  Outcomes: Depression (GDS-1, direct). |  |
| **Imprecision** | No  serious (-1)  very serious (-2) | Claims "very precise estimates" of possible effect, suggesting adequate power and that results (even if null) are precise. |  |
| **Publication Bias** | Undetected  Strongly suspected (-1) | Funded by NIHR HTA (Health Technology Assessment), which implies publication regardless of outcome, mitigating publication bias. |  |
| **Other**  (upgrading factors, circle all that apply) | Large effect (+1 or +2)  Dose response (+1 or +2) No Plausible confounding (+1 or +2) | No clear reasons to upgrade certainty. |  |
