# Supplementary material for: Effectiveness of physiotherapy techniques on depressive symptoms in older adults: a systematic review
Source: Front Public Health. 2025 Sep 17;13:1671788. doi: 10.3389/fpubh.2025.1671788 (PMC12483849; doi:10.3389/fpubh.2025.1671788)
Supplement: Supplementary file 2 [file Table_6.docx]

Supplementary File S6. Grade Summary

| **Reference** | **Outcome** | **Risk of Bias** | **Inconsistency** | **Indirectness** | **Imprecision** | **Publication Bias** | **Quality of the evidence** |
| --- | --- | --- | --- | --- | --- | --- | --- |
| **Aibar et al. (2019)** | **Depression** |  |  |  |  |  |  |
| **Conradsson et al. (2009)** |  |  |  |  |  |  |  |
| **Göksin et al. (2021)** |  |  |  |  |  |  |  |
| **Jung et al. (2022)** |  |  |  |  |  |  |  |
| **Neviani et al. (2017)** |  |  |  |  |  |  |  |
| **Penninx et al. (2002)** |  |  |  |  |  |  |  |
| **Singh et al. (1997)** |  |  |  |  |  |  |  |
| **Underwood et al. (2013)** |  |  |  |  |  |  |  |

Supplementary File S6. Grade Summary © 2025 by José Lesmes Poveda López is licensed under CC BY 4.0. To view a copy of this license, visit https://creativecommons.org/licenses/by/4.0/
